# Breast cancer susceptibility is associated with Cyclin D1 single nucleotide polymorphisms in Iran: A case-control study

**DOI:** 10.22099/mbrc.2025.51763.2065

**Published:** 2025

**Authors:** Sadaf Soleimani, Soheila Talesh Sasani, Zivar Salehi, Fereshteh Fakour

**Affiliations:** 1 Department of Biology, Faculty of Sciences, University of Guilan, Rasht, Iran; 2 Medicine School, Guilan Medical University, Rasht, Iran

**Keywords:** Cancer, Gene Regulation, Cycline D, Polymorphism, rs9344

## Abstract

Breast cancer (BC) is the main cause of cancer-related death in women worldwide. We evaluated the association between the key *CCND1* gene variant; rs9344 (G>A); and BC risk in Iran. In this case-control study, blood samples were obtained from 58 patients and 66 healthy controls. Genotyping was conducted by tetra-primer amplification refractory mutation system PCR (T-ARMS-PCR). Statistical analysis was performed by MedCalc software. Our results showed that the polymorphism rs9344 has an association with BC risk in the Iranian population. Based on the codominant and recessive models, carriers of the AA genotype are nearly 3.5 times more susceptible to BC than other individuals, and the AA genotype of *CCND1* A870G may be a significant factor for breast cancer. Further studies are needed to clarify the roles of *CCND1* polymorphism, rs9344, in breast cancer.

## INTRODUCTION

BC is the most common cancer among women and the second most prevalent worldwide [1]. BC is multifactorial; and influenced by demographic, reproductive, hormonal, hereditary, and lifestyle factors. Cyclins are proteins that aid cell cycle progression by activating CDKs [2]. Cyclin D1, encoded by the *CCND1* (MIM: 168461) on chromosome 11q13.14, regulates the G1/S phase transition; crucial for carcinogenesis initiation. The protein forms a phosphorylated complex with CDK4/CDK6, promoting the cell cycle by inactivating retinoblastoma protein. The relationship between *CCND1* and various cancers, including breast, colon, esophagus, and lung cancers, has been widely studied [3]. Single nucleotide polymorphisms (SNPs) are potential diagnostic and therapeutic biomarkers of many cancers. *CCND1* SNPs can lead to abnormal Cyclin D1 protein levels or dysfunction [4, 5]. G870A (rs9344) is a common *CCND1* polymorphism that results in a G→A substitution; linked to various cancer susceptibilities [4]. However, its role is unclear, with few studies on its association with cancer risk.

## MATERIALS AND METHODS

In this case-control study, 58 breast cancer patients and 66 healthy age-matched women (40-70 years) were enrolled. The study was approved by the Guilan Ethics Committee, Iran (Approval ID: GUILAN.REC.1400.03). Sampling was conducted at Golsar Hospital, Rasht, with informed consent. Clinicopathological histories were collected based on the American Joint Committee on Cancer Staging Manual. Blood samples were collected in ethylenediamine-tetraacetic acid (EDTA)-coated tubes and transferred to the Genetics Lab; University of Guilan, using ice packs. 

Genomic DNA was extracted from whole-blood samples using Triton-X100 (MERK, Germany). The purity and concentration of the extracted DNA were assessed by 1% agarose gel electrophoresis and a Nanodrop spectrophotometer (Thermo Fisher Scientific, Massachusetts, USA), respectively. Genotyping was carried out by tetra-primer amplification refractory mutation system PCR (T-ARMS-PCR). For genotyping the rs9344, 4 specific primers (F outer, F inner, R outer, R inner) were designed using Primer3 online software v4.1.0 and were analyzed by the NCBI Primer-BLAST tool (http://www.ncbi.nlm.nih.gov/). The special primers sequences were: F outer 5′-AAGTTCATTTCCAATCCGTCC-3′, R outer 5′-CCCAGAAACTC TACGGCTCTTG-3′, F inner (A allele) 5′-CAGAGTGATCAAGTGTGACGCA-3′, R inner (G allele) 5′-GGACATCACCCTCACTTGCC-3′. T-ARMS PCR was conducted in 25 μl volumes: 5 μl genomic DNA (70-100 ng/μl), 1 μl each of outer and inner specific primers (10 pmol/μl), 12 μl 2X Master mix red (Ampliqon, Denmark), and 5 μl dH_2_O. PCR was performed using a BioRAD MJ Mini Thermal Cycler. Conditions for rs9344: 95˚C for 5 min, 40 cycles of 95˚C for 30 s, 59˚C for 30 s, 72˚C for 30 s, and a final 72˚C for 8 min. Electrophoresis on 2% agarose gel was used to detect alleles by length.

Statistical analysis was performed by MedCalc (Version 17.9.7, Mariakerke, Belgium) software. Chi-square test were used for the Hardy-Weinberg equilibrium (HWE). The strength of association between the genotypes and the BC risk was estimated by odds ratio (ORs) and 95% confidence intervals (CIs).

## RESULTS AND DISCUSSION

The clinicopathological characteristics of the patients are shown in Table S1. We evaluated the association between rs9344 and breast cancer risk under different genetic models: co-dominant, dominant, recessive, and overdominant (Table 1 and Table S2). There was no significant difference between the observed and expected genotype frequencies in the control group ($\chi^{2}$=2.19, df=1, p=0.138). Our study showed that rs9344 was associated with breast cancer risk in Iranian population. Based on the co-dominant model, the AA genotype showed about three times higher susceptibility to breast cancer than the reference genotype. In the recessive model (AA vs. AG + GG), the AA genotype increased the risk of BC by almost 3.8 times.

**Table 1:** Association between the rs9344 and breast cancer risk

**Genotypes**	**Cases n (%)**	**Controls n (%)**		**OR**	**95% Cl**	**p-value**
**GG**	19 (32.76)	22 (33.34)		1.0	-	-
**AG**	21 (36.20)	37 (56.06)		0.65	0.29-1.48	0.657
**AA**	18 (31.04)	7 (10.6)		2.97	1.02-8.65	0.045
						
**GG**	19 (32.76)	22 (33.34)		1.0	-	-
**AG+AA**	39 (67.24)	44 (66.66)		1.02	0.48-2.17	0.946
						
**GG+AG**	40 (68.96)	59 (89.4)		1.0	-	-
**AA**	18 (31.04)	7 (10.6)		3.79	1.45-9.91	0.006
						
**GG+AA**	37 (63.8)	29 (43.94)		1.0	-	-
**AG**	21 (36.20)	37 (56.06)		0.44	0.21-0.91	0.028

**Note: **OR: Odds ratio, CI: confidence interval.

Our present study showed significant association between the rs9344 and the risk of BC There are two other published studies [5, 6] from Iran in this subjects, one of them supports our finding [6] and another one reported no association [5] which is contradictory to our finding. A meta-analysis found an association between rs9344 and breast cancer in Asian and European ancestry [7]. An association between rs9344 and colorectal cancer risk has also been reported from Iran [8].

Cyclin D1 regulates the cell cycle and can indirectly inhibit cell migration by preventing epithelial-mesenchymal transition (EMT). Thus, rs9344 as a functional polymorphism may affect the function of *CCND1* gene and may be an important biomarker for predicting breast cancer risk [9]. Taken together, these studies showed that *CCND1* rs9344 may be an important biomarker for predicting breast cancer risk. Studies on the association of the gene single nucleotide polymorphisms and cancer are affected by factors such as sample size, ethnicity, and environmental context, leading to different results. Therefore, further studies are needed to determine the exact role of *CCND1* rs9344 (G>A) in breast cancer.

## Data Availability

The datasets generated during the current study are available from the corresponding author upon reasonable request.

## Fundings:

This research did not receive a grant from any funding agency.
