# Supplementary material for: Breast cancer susceptibility is associated with Cyclin D1 single nucleotide polymorphisms in Iran: A case-control study
Source: Mol Biol Res Commun. 2025;14(3):199–202. doi: 10.22099/mbrc.2025.51763.2065 (PMC12046365; doi:10.22099/mbrc.2025.51763.2065)
Supplement: Supplementary file 1 — Tables S1-S2 [file MBRC-14-199-s001.pdf]

Table S1: Clinicopathological variables of Breast Cancer patients

Characteristic	Status	Number of patients n (%)	Number of controls n (%)
**Age (year)**	(<50)	33(56.9)	27(40.9)
	(>50)	25(43.1)	39(59.1)
**Tumor stage**	(0-II)	23(39.65)	0
	(III-IV)	35(60.35)	0
**Tumor size**	(<2 cm)	19(32.75)	0
	(>2 cm)	39(67.25)	0
**ER Receptor**	Positive	31(53.45)	N/A
	Negative	27(46.55)	N/A
**PR receptor**	Positive	28(48.27)	N/A
	Negative	30(51.73)	N/A
**HER2/neu receptor**	Positive	10(17.24)	N/A
	Negative	40(82.76)	N/A

N/A: Non-available

Table S2: Number and frequency of CCND1 A870G (rs9344) alleles

allele	Control (%)	Case (%)	Total (%)	χ^2	df	p-value
**A**	51 (38.64)	55 (48.25)	106 (43.09)	1.9282	1	0.165
**G**	81 (61.36)	59 (51.75)	140 (56.91)			

df: degrees of freedom
